# Physical Properties and Antimicrobial Release Ability of Gentamicin-Loaded Apatite Cement/α-TCP Composites: An In Vitro Study

**DOI:** 10.3390/ma16030995

**Published:** 2023-01-21

**Authors:** Kazuki Sasaki, Yoshiaki Ninomiya, Masaaki Takechi, Kanji Tsuru, Kunio Ishikawa, Hideo Shigeishi, Kouji Ohta, Tomonao Aikawa

**Affiliations:** 1 Department of Oral and Maxillofacial Surgery, Graduate School of Biomedical and Health Sciences, Hiroshima University, Hiroshima 734-8553, Japan; 2 Department of Dentistry, Oral and Maxillofacial Surgery, National Hospital Organization Kure Medical Center and Chugoku Cancer Center, Kure 737-0023, Japan; 3 Section of Bioengineering, Department of Dental Engineering, Fukuoka Dental College, Fukuoka 814-0193, Japan; 4 Department of Biomaterials, Faculty of Dental Science, Kyushu University, Fukuoka 812-8582, Japan; 5 Department of Public Oral Health, Program of Oral Health Sciences, Graduate School of Biomedical and Health Sciences, Hiroshima University, Hiroshima 734-8553, Japan

**Keywords:** apatite cement, alpha-tricalcium phosphate, hydroxyapatite, gentamicin

## Abstract

Apatite cement (AC), which has excellent osteoconductive ability, and alpha-tricalcium phosphate (α-TCP), which can be used for bone replacement, are useful bone substitute materials. The objective of this study was to clarify the physical properties and antimicrobial release ability of antibiotic-loaded AC/α-TCP composites in vitro. Gentamicin-loaded, rapid setting AC/α-TCP composites were prepared in 2 mixing ratios (10:3 and 10:6). The cement paste of AC/α-TCP composites was prepared in a plastic mold and dried in a thermostatic chamber at 37 °C and 100% relative humidity for 24 h. A diametral tensile strength test, powder X-ray diffraction analysis, and gentamicin release test were performed. The diametral tensile strengths of the AC/α-TCP composites were significantly less than that of AC alone. Powder X-ray diffraction patterns exhibited the characteristic peaks of hydroxyapatite in the AC/α-TCP composites and gentamicin-loaded AC/α-TCP composites. The concentration of the released gentamicin was maintained above the minimum inhibitory concentration of *Staphylococcus aureus* until Day 30 in both the gentamicin-loaded AC/α-TCP composites (10:3 and 10:6). Our results suggest that a gentamicin-loaded AC/α-TCP composite has potential as a drug delivery system. Further study is essential to investigate the antimicrobial activity and safety of the gentamicin-loaded AC/α-TCP composites in animal models.

## 1. Introduction

Osteomyelitis of the jaw is an intractable disease in the field of oral surgery [1]. Surgery is the common treatment; however, issues with transferring sufficient amounts of antimicrobial agents to the local lesions and regenerating bone tissue in surgical defects remain [2]. Therefore, bone grafting into the bone defect and local release of antimicrobial agents after surgical treatment are essential. Bone replacement materials containing antimicrobial agents in the bone resection area enable both the sustained release of antimicrobial agents and new bone formation in the bone defect.

Calcium phosphate cement is one of the most common bone replacement materials used in clinical practice. Calcium phosphate cements include apatite cement (AC), alpha-tricalcium phosphate (α-TCP), and beta-tricalcium phosphate (β-TCP), each of which has different characteristics in terms of osteoconductivity and dissolution. Conventional AC was developed by Brown and Chow in 1986, and it has excellent biocompatibility and osteoconductivity [3]. An in vitro analysis using mouse osteoblastic cells revealed that AC containing atelocollagen has a high osteoconductivity [4]. However, one of the problems of conventional AC is its long cure time (i.e., 30–60 min) [5,6,7,8,9,10,11,12]. Ishikawa et al. investigated the setting mechanism of AC and developed a rapid set AC with shorter setting time (i.e., 5 min), which is now widely used clinically [13]. Therefore, rapid set AC has excellent operational efficacy in clinical sites where rapid setting of the cement is required. Although AC is replaced by bone, it generally takes a long time for complete bone replacement to occur [7,8,10,11,12]. Therefore, AC has inferior osteoinductive properties. In comparison, α-TCP has similar physical properties and biocompatibility and, furthermore, has the highest solubility among the calcium phosphate cements [14], making it likely to be more rapidly replaced by new bone. However, α-TCP has inferior space-making ability for new bone formation.

A composite of AC with excellent osteoconductive ability and α-TCP with high solubility and excellent bone replacement capacity is a useful replacement material for bone defects. Additionally, a composite of AC and α-TCP with the capacity to release antibiotics can be employed in bone defects after surgical treatment of osteomyelitis of the jaw. However, the physical properties of a composite of AC and α-TCP remains unknown. Therefore, we first prepared a composite of fast-setting AC and α-TCP.

Gentamicin is an amide glycoside with a broad antimicrobial spectrum, including *Pseudomonas aeruginosa*, and thermal resistance [15]. Gentamicin preparations are generally available in intravenous, ophthalmic, and topical forms. Therefore, gentamicin is widely employed for the treatment of infectious diseases. Gentamicin-loaded polymethylmethacrylate-based cement with a reduced liquid/powder ratio, increased radiopacifier ratio, and high antibiotic dose showed excellent gentamicin release in an in vitro analysis [16]. The implantation of gentamicin/vancomycin-loaded hydroxyapatite was effective for the treatment of chronic osteomyelitis of the tibia [17]. In addition, gentamicin/vancomycin-loaded calcium sulfate-hydroxyapatite composites were employed for the treatment of osteomyelitis of cuboid bone [18]. Thus, gentamicin is commonly employed for the preparation of antibiotic-loaded bone cement. Therefore, gentamicin-loaded composites of AC and α-TCP were prepared, and their physical properties and antimicrobial release ability in vitro were examined. The objective of this study was to clarify the physical properties and antimicrobial release ability of antibiotic-loaded AC/α-TCP composites.

## 2. Materials and Methods

### 2.1. Preparation of Gentamicin-Loaded AC/α-TCP Composites

**Figure 1:**
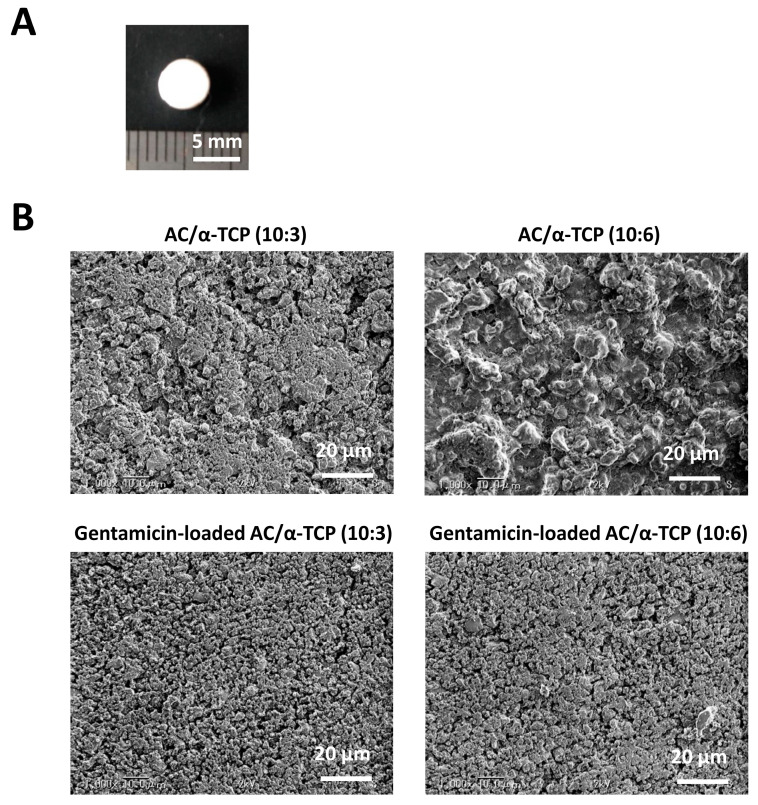
Preparation of gentamicin-loaded AC/α-TCP composites. (**A**) Photograph of the gentamicin-loaded AC/α-TCP composite. (**B**) SEM images of the AC/α-TCP and gentamicin-loaded AC/α-TCP composites.

The powder phase of AC, an equimolar mixture of dicalcium phosphate anhydrous and tetracalcium phosphate, was prepared in accordance with a previous study [4]. α-TCP was obtained from Taihei Chemical (Osaka, Japan). The mixing ratio of AC/α-TCP composites was 10:3 or 10:6. In addition, gentamicin sulfate salt powder (Sigma Aldrich, Saint Louis, MO, USA) was added to AC/α-TCP (10:3) and AC/α-TCP (10:6) at 10% by weight. Cement and gentamicin were mixed with 0.2 M neutral sodium phosphate buffer solution at a ratio of 1:3.5. The cement paste was placed in a plastic mold with a height of 2 mm and a diameter of 5 mm and then dried in a thermostatic chamber at 37 °C with 100% relative humidity for 24 h. Photographs of the gentamicin-loaded AC/α-TCP composites are shown in Figure 1A. The setting times of the cements were measured in a humidity chamber at 37 °C using a Vicat needle (MIC-307-1-01, MARUI & Co., Ltd., Osaka, Japan) and a 300 g needle with a diameter of 2.0 mm.

### 2.2. Morphological Observation by Scanning Electron Microscopy

Morphological observations were conducted using a scanning electron microscope (SEM, VE-8800, Keyence, Osaka, Japan). Gold was deposited on the surface of the specimen by an ion sputtering method, and SEM observation was conducted under an accelerated voltage of 15 kV.

### 2.3. Measurement of the Mechanical Strength

The diametral tensile strength was measured as an index of the mechanical strength of the gentamicin-loaded AC/α-TCP composites. The strength was measured using a small universal testing machine (AUTOGRAPH AGS-J, Shimadzu, Kyoto, Japan) at a crosshead speed of 1 mm/min.

### 2.4. Powder X-ray Diffraction Analysis

The compositions of the cured materials were analyzed using a powder X-ray diffraction system (D8 Advance, Bruker AXS Gmb, Karlsruhe, Germany). Gentamicin-loaded AC/α-TCP composites were analyzed by a powder X-ray diffractometer under the following conditions: tube voltage 40 kV, tube current 100 mA, Cu as target, and Ni as the filter. Phase analysis was performed using Powder Diffraction File (PDF) card No. 09-0432 for hydroxyapatite and No. 09-0348 for α-TCP.

### 2.5. Gentamicin Release Test

The antibacterial effect of gentamicin against *Staphylococcus aureus* 209P was examined. Gentamicin was diluted 2-fold with distilled water from 2048 µg/mL to 0.5 μg/mL. The diluted gentamicin was dropped onto Tryptic soy agar plates coated with *S. aureus* (1.0 × 10^6^ colony forming units). The diameter of the inhibition zone produced by gentamicin was measured after 24 h, and the antibacterial effect was presented as zones of inhibition. The relationship between the diameter of the inhibition zone and various concentrations of gentamicin was examined to create a standard curve.

Next, gentamicin-loaded AC/α-TCP composites were immersed in 200 µL of PBS at 37 °C for 24 h, and then 10 µL of the solution was dropped onto the agar plate coated with *S. aureus*. After 24 h of incubation, the diameter of the inhibition zone was measured from Day 1 to Day 30. The total volume of the PBS solution was changed every 24 h. The amount of antimicrobial release was calculated using a standard curve. Experiments were performed in triplicate and the results presented as mean ± standard deviation.

### 2.6. Statistical Analysis

Tukey’s test was used for multiple comparisons. *p* values < 0.05 were regarded as statistically significant.

## 3. Results

### 3.1. SEM Observation and Setting Time of the Gentamicin-Loaded AC/α-TCP Composites

**Figure 2:**
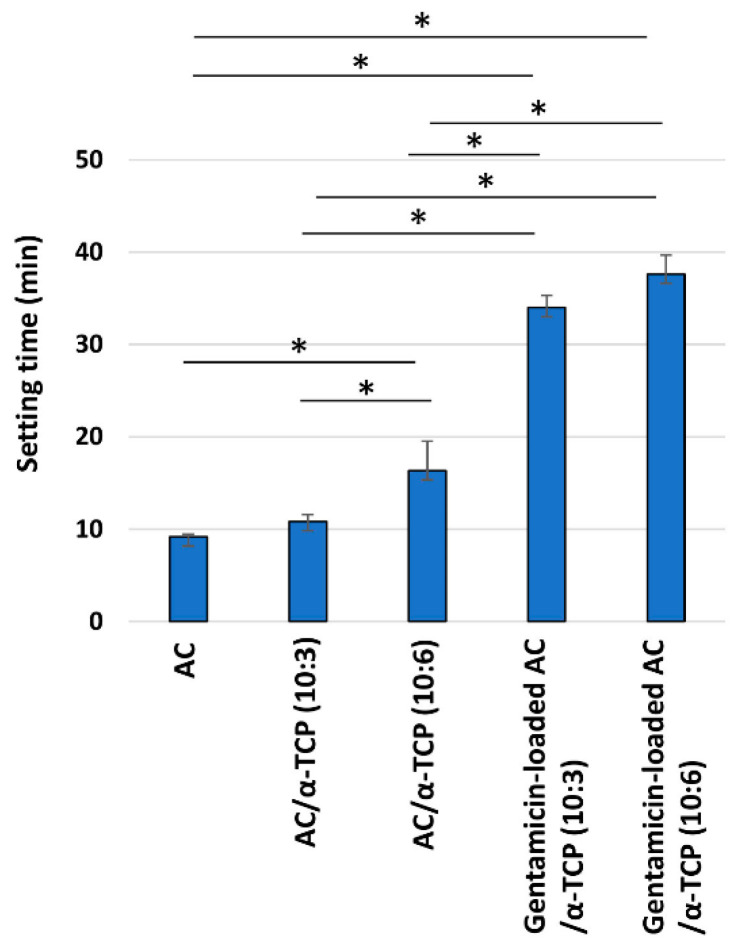
Setting time of gentamicin-loaded AC/α-TCP composites. * *p* < 0.05, Tukey’s test.

SEM images of the surface of AC/α-TCP composites and gentamicin-loaded AC/α-TCP composites are shown in Figure 1B. AC/α-TCP composites without antimicrobial agent showed a rough surface and dense crystal structure. AC/α-TCP (10:6) had both large and small α-TCP particles. In contrast, gentamicin-loaded AC/α-TCP (10:3) and gentamicin-loaded AC/α-TCP (10:6) showed gentamicin particles precipitated on the surface of the hardened cements. Setting time was significantly longer in AC/α-TCP (10:6) than AC, while gentamicin-loaded AC/α-TCP composites (10:3 and 10:6) respectively showed longer setting times than AC/α-TCP composites (10:3 and 10:6) alone (Figure 2).

### 3.2. Mechanical Strength of the Gentamicin-Loaded AC/α-TCP Composites

**Figure 3:**
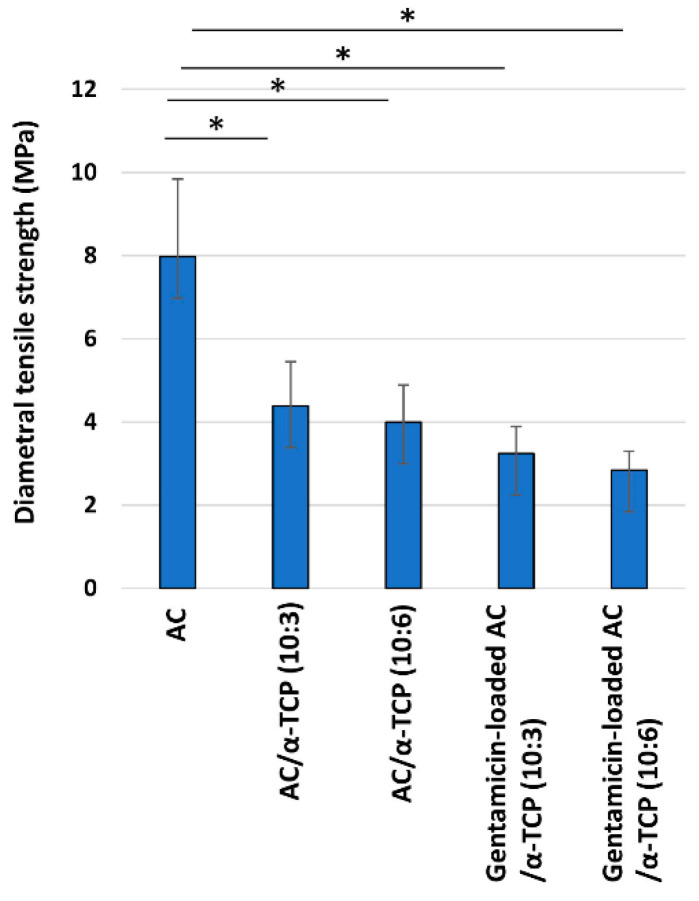
Diametral tensile strength of the AC/α-TCP and gentamicin-loaded AC/α-TCP composites. * *p* < 0.05, Tukey’s test.

The diametral tensile strength of the AC/α-TCP and gentamicin-loaded AC/α-TCP composites are shown in Figure 3. The diametral tensile strengths of AC/α-TCP (10:3) and AC/α-TCP (10:6) were significantly less than that of AC alone. Gentamicin-loaded AC/α-TCP (10:3) and gentamicin-loaded AC/α-TCP (10:6) exhibited a significantly lower diametral tensile strength than AC alone. Gentamicin loading further reduced the diametral tensile strength of the AC/α-TCP composites (though less significantly). In addition, there was no significant difference in the diametral tensile strength between AC/α-TCP (10:3) and AC/α-TCP (10:6) or between gentamicin-loaded AC/α-TCP (10:3) and gentamicin-loaded AC/α-TCP (10:6).

### 3.3. Powder X-ray Diffraction Analysis of the Gentamicin-Loaded AC/α-TCP Composites

**Figure 4:**
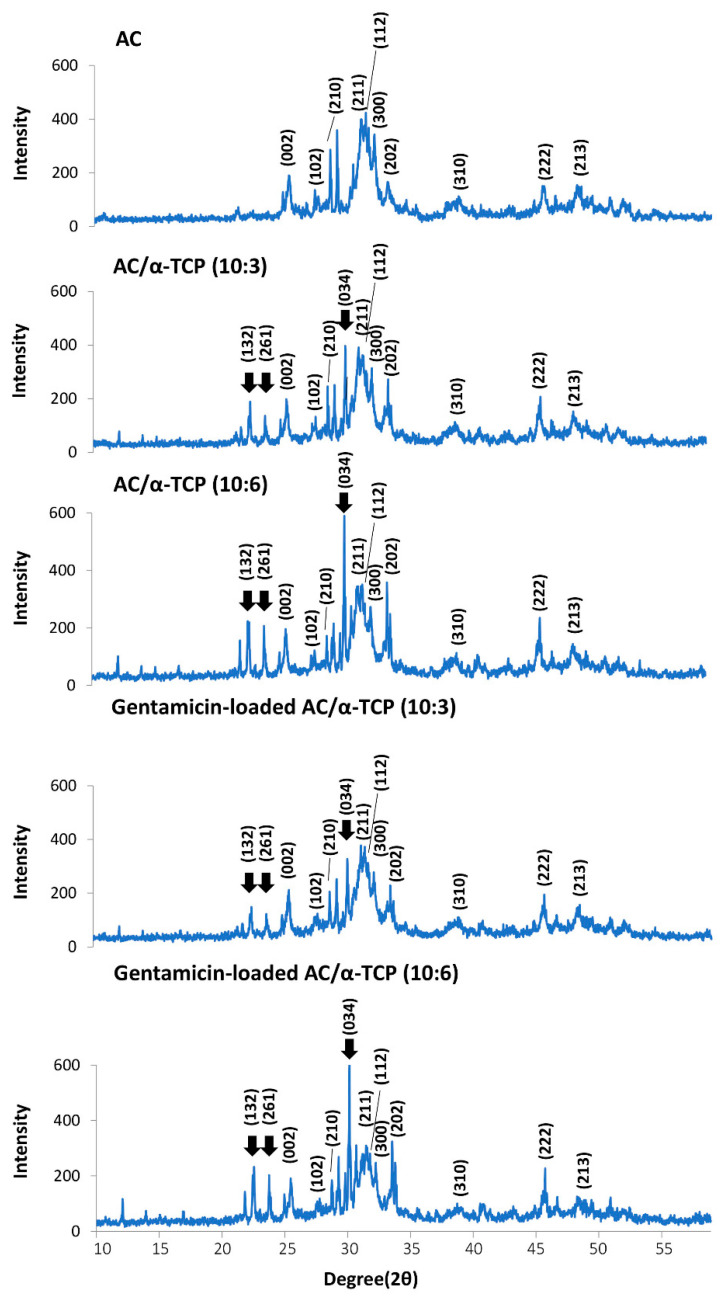
Powder X-ray diffraction patterns of the AC/α-TCP and gentamicin-loaded AC/α-TCP composites. Peaks of hydroxyapatite at 25.9°, 28.3°, 29.3°, 31.8°, 32.2°, 32.9°, 34.0°, 39.8°, 46.7°, and 49.5° were indexed as (002), (102), (210), (211), (112), (300), (202), (310), (222), and (213), respectively. Peaks of α-TCP at 22.8°, 24.2°, and 30.7° were indexed as (132), (261), and (034), respectively. (arrow).

The results of the powder X-ray diffraction analysis are shown in Figure 4. AC, AC/α-TCP (10:3), and AC/α-TCP (10:6) showed powder X-ray diffraction peaks at 25.9°, 28.3°, 29.3°, 31.8°, 32.2°, 32.9°, 34.0°, 39.8°, 46.7°, and 49.5°, which are characteristic of hydroxyapatite crystals [19]. Additionally, peaks at 25.9°, 28.3°, 29.3°, 31.8°, 32.2°, 32.9°, 34.0°, 39.8°, 46.7°, and 49.5° were observed in both gentamicin-loaded AC/α-TCP (10:3) and gentamicin-loaded AC/α-TCP (10:6). These results suggest that the AC in the AC/α-TCP and gentamicin-loaded AC/α-TCP composites was converted to hydroxyapatite. Major peaks of α-TCP at 22.8°, 24.2°, and 30.7° were observed in the AC/α-TCP composites and gentamicin-loaded AC/α-TCP composites.

### 3.4. Antimicrobial Release of the Gentamicin-Loaded AC/α-TCP Composites

**Figure 5:**
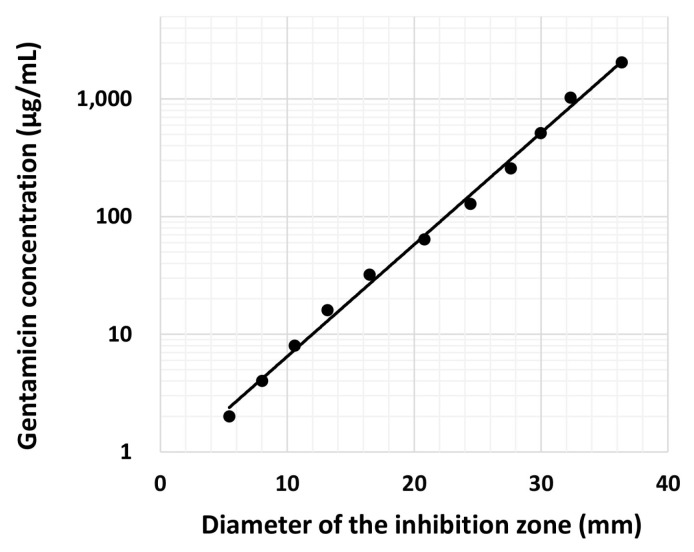
Relationship between gentamicin concentrations and the diameters of the inhibition zone in an agar plate coated with *Staphylococcus aureus*.

**Figure 6:**
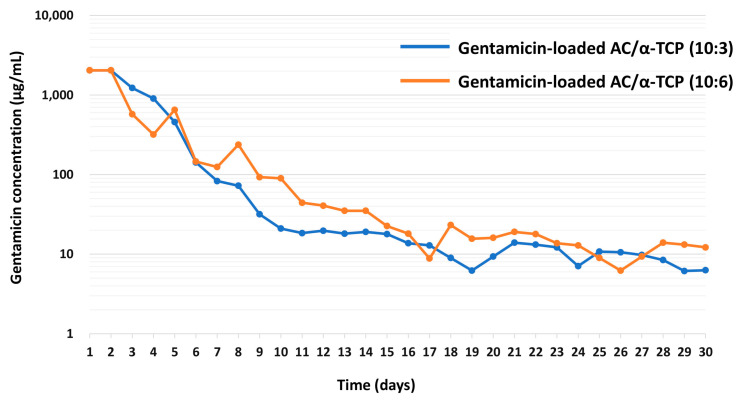
The concentration of gentamicin released from gentamicin-loaded AC/α-TCP composites.

Standard curve for gentamicin concentrations versus the diameters of the inhibition zone are shown in Figure 5. The minimum inhibitory concentration (MIC) of gentamicin was 2.0 μg/mL. Next, the concentrations of gentamicin released from gentamicin-loaded AC/α-TCP composites were calculated using a standard curve. The release of gentamicin from gentamicin-loaded AC/α-TCP composites from Day 1 to Day 30 is shown in Figure 6. The concentration of released gentamicin gradually decreased in both gentamicin-loaded AC/α-TCP (10:3) and gentamicin-loaded AC/α-TCP (10:6); however, the concentration was maintained above the MIC (2.0 μg/mL) of *S. aureus* until Day 30.

## 4. Discussion

Hydroxyapatite is a biomaterial widely used as a bone substitute that is considered a non-biodegradable material that remains where it is grafted [20]. Additionally, it is difficult to fit the hydroxyapatite shape to the bone defect [21]. In contrast, AC has an advantage because it can form hydroxyapatite after setting from a liquid and powder mixing phase [22]. However, blood and bodily fluids sometimes impair the hardening of AC because of its long setting time. Importantly, the setting time has been dramatically improved with fast-setting AC [14]. In addition, fast-setting AC has greater mechanical strength, and can transform to apatite-type phase faster than conventional AC [14]. Therefore, fast-setting AC has numerous advantages as a substitute for reconstruction after bone resection compared with conventional AC.

In this study, we found that the addition of α-TCP and gentamicin extended the setting time of AC. It has also been reported that added α-TCP affects the hardening and apatite crystal formation of AC [23]. The setting time of gentamicin-loaded AC/α-TCP (10:6) was within 40 min, indicating the relatively short setting time of gentamicin-loaded AC/α-TCP composites. Gentamicin-loaded AC/α-TCP composites had longer setting times than the AC/α-TCP composites. The acidic gentamicin sulfate salt solution may have inhibited the setting, extending the setting time of the AC/α-TCP composites.

AC/α-TCP composites showed less diametral tensile strength than the AC alone. The reduction is mainly attributed to the low diametral tensile strength of α-TCP. However, AC/α-TCP (10:3) and AC/α-TCP (10:6) exhibited similar diametral tensile strength, suggesting that varying the composite ratios of AC/α-TCP composites has little bearing on diametral tensile strength. Additionally, a significant reduction in the diametral tensile strength was not observed after gentamicin loading of the AC/α-TCP composites. Importantly, powder X-ray diffraction analysis revealed the conversion to hydroxyapatite in AC/α-TCP composites with or without gentamicin loading as well as in AC alone. The addition of gentamicin does not affect the conversion of AC to hydroxyapatite. It is likely that antimicrobial agent loaded AC/α-TCP composites maintain similar physical properties to their corresponding AC/α-TCP composites.

The sustained release of antimicrobial agents from bone grafting materials depends on the porosity of hydroxyapatite and the solubility of the antimicrobial agents [24]. Suzuki et al. performed an in vitro analysis to investigate the sustained release of antimicrobial agents from pastes with different antibiotics. The total amount of eluted antibiotics was 58% vancomycin, 47% gentamicin, and 2.1% flomoxef sodium, suggesting that the release is different for each antibiotic [25]. Importantly, high and sustained release of gentamicin from bone grafting materials was found [25]. The results of our in vitro analysis highlight the importance of gentamicin for a drug delivery system.

Continuous release of gentamicin from gentamicin-loaded AC/α-TCP was observed in this study. Importantly, the sustained release of gentamicin exceeded the MIC of *S. aureus* for a long period of 30 days, suggesting that gentamicin-loaded AC/α-TCP composites have continuous antimicrobial activity over 30 days. Our results indicate that gentamicin-loaded AC/α-TCP composites can be used as a drug delivery system and may be used in the treatment of patients with osteomyelitis. However, it remains unknown whether gentamicin-loaded AC/α-TCP composites have an antimicrobial ability in vivo. It is necessary to investigate the antimicrobial activity and safety of gentamicin-loaded AC/α-TCP composites in animal models. Additionally, there was no clear difference in gentamicin release ability between gentamicin-loaded AC/α-TCP (10:3) and (10:6). It is speculated that dissolution character of the AC/α-TCP composites is associated with the gentamicin release. However, dissolution of AC/α-TCP composites (10:3) and (10:6) remains unknown. Further studies are needed to clarify why the ratio between AC and α-TCP does not affect the release of gentamicin.

There were several limitations in this study regarding the characterization of the physical properties of AC/α-TCP composites. AC/α-TCP composites without gentamicin had a non-uniform crystal structure. Although it is important to investigate the distribution of aggregates according to the size of the AC/α-TCP composites, it was not possible in this study. In addition, the porosity of AC/α-TCP composites remains unknown. Furthermore, a powder X-ray diffraction analysis before conversion to the apatite phase was not performed. Therefore, it was impossible to compare the powder X-ray diffraction patterns before and after conversion.

Although the side effects and antibacterial effect of gentamicin-loaded AC/α-TCP composites on animal cells remain unknown, it is speculated that gentamicin-loaded AC/α-TCP composites have an acceptable biocompatibility and long-term antimicrobial activity. Therefore, there is the possibility that gentamicin-loaded AC/α-TCP composites can be employed as a local drug delivery system for the treatment of osteomyelitis. However, long-term administration of antibiotics may lead to the emergence of antibiotic resistant strains of bacteria. In contrast, recent studies revealed that nanoparticles, such as metallic nanoparticles and curcumin nanocrystals, have excellent antimicrobial capacity and therefore can be used for the treatment of multidrug-resistant bacterial strains [26,27,28,29]. Bone cements containing nanoparticles may be applicable to the treatment of osteomyelitis in the future.

Gap junctions are intercellular channels involved in the regulation of cell growth and differentiation between adjacent cells [30]. Therefore, gap junctions play an important role in osteoblast differentiation and bone formation [30]. Apatite cement-based scaffolds may impact the gap junction-mediated cell-to-cell communication in osteoblasts. However, the effect of gentamicin-loaded apatite cement/α-TCP composites on the cell-to-cell communication in osteoblasts remains unknown. Furthermore, it is speculated that there is a complex interaction between antibiotic administration and mitochondria dysfunction [31]. However, the impact of antibiotics on mitochondria function remains unclear in osteoblasts. Molecular investigation is necessary to clarify the association between gentamicin-loaded AC/α-TCP composites and mitochondria function as well as the gap junction-mediated communication between contiguous cells.

## 5. Conclusions

We prepared gentamicin-loaded AC/α-TCP composites for the purpose of new bone replacement and long-term antimicrobial agent release. Gentamicin-loaded AC/α-TCP composites have longer setting times than AC/α-TCP composites. Gentamicin-loaded AC/α-TCP composites have physical properties (i.e., diametral tensile strength and conversion into hydroxyapatite) that are similar to their corresponding AC/α-TCP composites. Importantly, gentamicin-loaded AC/α-TCP composites have continuous antimicrobial activity over a long period of time. The results of this study suggest that AC/α-TCP composites can be used as a novel bone cement for antibiotic delivery in future studies. Implantation of gentamicin-loaded AC/α-TCP composites may be an effective treatment after the surgical resection of jaw bones in the treatment of osteomyelitis. Further study is essential to clarify the biocompatibility, osteoconductive ability, and antimicrobial impact of gentamicin-loaded AC/α-TCP composites in vivo.

## Data Availability

All data generated or analyzed in this study are included in this article.
